# Making Choices in Discourse: New Alternative Masculinities Opposing the “Warrior’s Rest”

**DOI:** 10.3389/fpsyg.2021.674054

**Published:** 2021-05-25

**Authors:** Laura Ruiz-Eugenio, Ana Toledo del Cerro, Jim Crowther, Guiomar Merodio

**Affiliations:** ^1^Department of Theory and History of Education, University of Barcelona, Barcelona, Spain; ^2^Department of Sociology, University of Barcelona, Barcelona, Spain; ^3^Moray House School of Education and Sport, IECS, University of Edinburgh, Edinburgh, United Kingdom; ^4^Department of Education, Nebrija University, Madrid, Spain

**Keywords:** warrior’s rest, double standards, communicative acts, new alternative masculinities, the language of desire

## Abstract

Psychology research on men studies, attractiveness, and partner preferences has evolved from the influence of sociobiological perspectives to the role of interactions in shaping election toward sexual–affective relationships and desire toward different kinds of masculinities. However, there is a scientific gap in how language and communicative acts among women influence the kind of partner they feel attracted to and in the reproduction of relationship double standards, like the myth of the “warrior’s rest” where female attractiveness to “bad boys” is encouraged or supported. Some women imitate “the warrior” behavior of men by choosing dominant traditional masculinities (DTM) to have “fun” with and oppressed traditional masculinities (OTM) for “rest” after the “fun” with DTM—choosing an OTM for a stable relationship, but perhaps without passion, while also feeling attraction toward DTM, a response which perpetuates the chauvinist double standard that the feminist movement has condemned when men behave in this sexist way. Through conducting a qualitative study with communicative daily life stories, this article explores, on the one hand, how language and social interaction among women can lead to the reproduction of the DTM role by women and, on the other hand, also how new alternative masculinities (NAM) offer an alternative by explicitly rejecting, through the language of desire, to be the rest for the female warrior, the second fiddle to any woman. This has the potential to become a highly attractive alternative to DTM. Findings provide new knowledge through the analysis of communicative acts and masculinities evidencing the importance of language uses in the reproduction of the double standards in gender relations and to understand how and why these practices are maintained and which kind of language uses can contribute to preventing them. Implications for research and interventions on preventive socialization of gender violence are discussed.

## Introduction

The women’s movement and other progressive social movements have traditionally been committed to foster gender equality by condemning, among other issues, gender inequalities regarding sexual behavior and interpersonal relationships by advocating sexual freedom and equality. Therefore, double standards about social attitudes and expectations toward the sexual behavior of men and women and its consequences in terms of gender differences have been widely examined and challenged (Kreager and Staff, 2009; Lyons et al., 2011; Dunn et al., 2014; Zaikman and Marks, 2014; Rios-González et al., 2018; Armstrong et al., 2020; Duque et al., 2020; Kim, 2020; Álvarez-Muelas et al., 2021). Feminist theories (Beauvoir, 1949; Greer, 1970) have condemned the sexist myth of the *warrior’s rest* that positions women as passive sexual objects, subject to their partners’ dominance and dependence. This patriarchal myth relies on the double standard and classifies women into two types, those for casual sex and those for marital and family purposes. The *warrior’s rest* practices are exerted by dominant traditional masculinities (DTM), which have been traditionally linked with a successful image of aggressive and chauvinist manhood (Flecha et al., 2013). Nevertheless, some women also imitate the *warrior’s rest* by reproducing these chauvinist double standards (Valls et al., 2008; Lyons et al., 2011; Gómez, 2015). However, this role reversal does not foster women’s sexual agency, quite the opposite. It perpetuates traditional double standards and gender inequality.

While this phenomenon has been analyzed in gender studies, it remains unexplored through communicative acts and men studies’ prism. This article addresses this, firstly, by conducting a qualitative study on how language and social interaction among women can lead to the reproduction of appreciation and esteem that the DTM role receives by women and, secondly, by examining how new alternative masculinities (NAM) can become a highly attractive alternative to DTM when they use the language of desire for explicitly rejecting to be the rest for the female *warrior*. The language of desire is a language connected to attraction, tastes, and sexual excitement (Flecha and Puigvert, 2010; López de Aguileta et al., 2020). In order to address these themes, we firstly present a selection of scientific literature on sexual double standards and gender role reversal, as well as the influence of social interactions and language uses on women’s partner choice and the role of NAM with particular attention to the role of communication in this regard. Secondly, we describe the methods of the qualitative study which was conducted. The study results are presented in two parts: language and interaction among a group of women friends that promote the imitation of the *warrior’s rest* by women and how some communicative acts by men can manage to overcome this tendency. Finally, we end with a discussion of the results and conclusions for future research as well as their implication for gender violence prevention.

### Reversing Traditional Roles and Perpetuating the Double Standard

Psychology research on men studies, attractiveness, partner preferences, and gender differences in partner choice has traditionally focused on the influence of different elements such as sociobiological aspects, hormones and pheromones, facial appearance, sexual dimorphism, physical attributes, and body shape preferences (Perilloux et al., 2013; Price et al., 2013; Wells et al., 2013; Hatz et al., 2020; White et al., 2020). Other studies have analyzed the influence of interactions and peer groups in selecting sexual affective relationships and the desire toward different kinds of masculinities (Herold and Milhausen, 1999; Urbaniak and Kilmann, 2003; McDaniel, 2005). On this subject, most studies draw from psychology and sexology. However, there is a significant gap in scientific knowledge about the influence of interactions, language, and communicative acts among women that influence them to choose and initiate sexual relationships with DTM and to imitate a female version of the traditional *warrior’s rest* or alternatively that promote, through the language of desire, the rejection by NAM to be the rest for the female *warrior*.

Since the 1980s, researchers have noted the sexual double standard that influences men’s and women’s sexual behavior by penalizing women, being less permissive for them, and alternatively forgiving or rewarding men for the same sexual behavior (Lyons et al., 2011; McClintock, 2011; Dunn et al., 2014; Zaikman and Marks, 2014; Pecheny et al., 2019; Álvarez-Muelas et al., 2021). In this regard, Manago et al. (2015) examined in a study the double standards in the messages about sex that adolescents receive from their friends, showing how girls were conveyed more values linked to relational sex and boys to recreational sex. Nowadays, the traditional double standard persists, with reports of worse consequences for girls, who suffer from their reputation being harmed, especially in school contexts (Amaro et al., 2020). Lyons et al. (2011) analyzed the meaning of the sexual double standard at the peer level, gathering data through interviews conducted with female students where some of them reverse the traditional roles and imitate the double standard of men. They also highlighted the kind of adjectives and conversations that often emerge between peers when women reproduce the traditional role. For instance, usual adjectives are employed to define them such as “slut,” and repeated “behind the back” conversations of a harmful kind commonly occur. In this regard, other research has also evidenced the influence of social imaginaries and media messages, often promoted by romantic novels or in women’s erotic literature, that perpetuate this reversal of roles (Hawley and Hensley, 2009). Research also suggests that friendship among girls during their adolescence can either protect them from their peer critics regarding their sexual behavior or lead to double standard reproduction (Racionero-Plaza et al., 2021).

The double standards are also linked to masculinity models and partner choice. In a study conducted by Tifft (2015), women interviewed regarding their sexual interest classified men into two different groups: “nice boys,” where the language used to describe men is connected with care and ethical behavior such as “giving” and “caring,” or in terms of “bad boys,” where more aggressive words are used such as “rude” and “dominant.” Some of the reason that explains this duality will be examined in more detail in the next section by analyzing social interaction and its influence on women’s desire.

### Influence of Social Interactions on Women’s Attraction to DTM or Oppressed Traditional Masculinities (OTM)

Regarding women’s attraction and influences on partner choice, research has explored the repercussions of social and cultural contexts, such as romantic literature, on the influence on women’s choices and relationship models (Barros del Río, 2005). Beyond the importance of these elements, the influence of social networks and friendship on romantic relationships and partner choice during the start of the relationship and throughout the relationship is clearly crucial (Wright and Sinclair, 2012; Killoren et al., 2019; Elboj-Saso et al., 2020). Also, research on the language use emphasizes the potential of language in socialization processes (Burdelski, 2013; Rios-González et al., 2018; Arnold, 2019) and in the transmission of patterns of gender inequalities among children (da Luz Scherf et al., 2020). Regarding partner choice, other researchers report that young girls are more influenced by the popularity and prestige of their potential partners (Little et al., 2015; Gouda-Vossos et al., 2018). In addition, there are also studies that analyze if women prefer more or less typically masculine men and the influence of gender equality and traditional gender norms on this selection (Desrochers, 1995). However, such studies have not paid much attention to the influence of the interactions, language, and communicative acts among women toward the kind of partner they feel attracted to and its influence in perpetuating double standards enacted by men and women.

Other research has highlighted the duality through which “nice guys” are more desirable for committed relationships but are perceived as less sexually successful in contrast to those considered as “not nice” (Herold and Milhausen, 1999). In this regard, Urbaniak and Kilmann (2003) explored the nice guy stereotype and the pejorative discourse around the “nice guys finish last,” which has become a motto to define these heterosexual men unsuccessful in sex because they do not treat women very badly. Ahmetoglu and Swami (2012) have also examined how some women defined men who looked dominant versus those known as “nice guys” as more sexually attractive. As a consequence of this discourse, most women recommended the nice guys as the best choice for having more serious, long-term, and stable relationships, while they preferred a “macho man” for casual and sexual relationships. In the same line, McDaniel (2005) examined the duality and double standard that girls experience regarding the choice and attraction to men exhibiting different masculinities. Thus, this author points out that some girls have shown a desire toward romantic and stable relationships with “nice guys” but nevertheless prefer to have sexual relationships with “jerks” and “bad guys.” In fact, these young women describe “nice guys” with adjectives that indicate the absence of desire, such as “someone my friends would like.” Sometimes, the bad guys are referred to in terms that mix desire with violent connotations such as “physically attractive” and “aggressive.” Although it was not a specific dimension of the study, it would have been interesting to analyze the kind of language that is behind each conception and desirable relationship. More evidence in this regard was illustrated by Georgakopoulou (2005) in her analysis of young heterosexual women dialogues about guys. She discovered how these conversations reinforce specific male images labeling “soft men” with humorous references like “baby face.”

The line of research on preventive socialization of gender violence provides evidence of the influence of the dominant coercive discourse in partner preferences and desire that perpetuates double standards and associates attraction with violence (Gómez, 2015; Elboj-Saso et al., 2020). The quantitative study of Puigvert et al. (2019) with high school girls showed female participants’ preference for boys with violent attitudes for sporadic relationships and “hooking up” and for non-violent boys for stable relationships. This reproduces double standards by presenting violent and unequal relationships as exciting and egalitarian relationships as convenient (Torras-Gómez et al., 2020). In short, scientific literature confirms the duality in the selection and attractiveness between both masculinities, the “jerks” and the “nice guys,” which perpetuates the traditional model of the double standard because while the objective is to get a good, perfect guy to have a stable relationship with, young women’s attraction to “jerks” and “bad guys” is seen to be more sexually exciting (Puigvert et al., 2019). Some analysis goes beyond and evidences that the duality between both masculinities persists because they complement each other, reproducing the traditional chauvinist double standards (Flecha et al., 2013; Díez-Palomar et al., 2014).

### NAM Reject Perpetuating the Double Standard Through the Language of Desire

Research on the use of language and social interactions among men has focused mainly on gender differences between men and women, the topics of the conversation, the gendered use of language, the influence of social context on interpersonal communication (Stobbe, 2005; Portell and Pulido, 2012; Rodríguez-Navarro et al., 2014), the behavior and communication between men in specific contexts such as fraternities (Fabius, 2005), and the importance of friendship on their masculinity (Gómez, 2014; Migliaccio, 2014; Boulton, 2020). Regarding the manifestation of the linguistic process, research has also pointed out the different interactional styles between men and women (Baxter, 2006; Jackson, 2018). Other studies have analyzed the use of non-verbal signs and communication behavior among men on partner selection and courtship interaction (Renninger et al., 2004; Brak-Lamy, 2015; Fisher et al., 2020). Besides, many researchers have related the accounts of violence and the discourse of hegemonic masculinity (Connell, 1995; Mullaney, 2007; Mañas-Viejo and Martínez Sanz, 2020; Mensah, 2021).

In short, there is a gap in the study of exclusionary language that perpetuates the double standard. On the one hand, there is a dearth of literature about the language used by the NAM (Portell and Pulido, 2012; Rodríguez-Navarro et al., 2014), and on the other hand, there are no studies that connect this discourse with the myth of the *warrior’s rest*. In this regard, the aforementioned duality in the selection between DTM and OTM omits another kind of alternative masculinity (Connell, 1987; Bridges and Pascoe, 2014), the NAM, where feelings of passion and respect come together in the same person. As Flecha et al. (2013) concluded, NAM’s distinctive traits are the attractiveness and desire that they generate; and particularly, this happens due to the verbal and body language which they employ that include an emphasis on desire and equality at the same time. This is an aspect that contributes to the attraction of an alternative masculinity model (NAM).

Regarding the potentiality of speech and communicative acts for transforming reality, Fairclough (2003, 2006) highlighted that speech not only is necessary to shape and preserve social structures but also helps to challenge and transform them. Even though cultural discourses and social interactions often influence men and women, they are at the same time active participants in the discourses’ development, reinscription, or rewriting (Fabius, 2005). Therefore, cultural and social discourses can be argued, and previous ideas can change, which is an important aspect of partner choice and relationships (Giordano et al., 2006). Social interactions, dialogues (Austin, 1962; Habermas, 1987; Searle, 1998), and communicative acts that include not only language but also gestures and the tone of voice as well as the social context (Searle and Soler, 2004; Flecha et al., 2020) are fundamental in transforming the processes that contribute or challenge sexual double standards. Moreover, research has already shown that communicative acts linked to NAM are decisive when preventing gender violence (Portell and Pulido, 2012; Rodríguez-Navarro et al., 2014).

## Materials and Methods

### Methodological Approach

Two questions have guided the present research: *How do language and social interactions among women lead to the reproduction of and esteem that the DTM role receives by some women? How can NAM, through the language of desire, resist the female warrior’s double standards?* To answer these questions, the purpose of the study was two-fold: (a) to analyze how language and social interaction among women can lead to the reproduction of DTM role by women and (b) to explore how NAM offer an alternative by explicitly rejecting, through the language of desire, to be the rest for the female *warrior*. The present research followed the communicative methodology (CM) approach (Gómez et al., 2011, 2012, 2019; Flecha, 2014; Gómez, 2017; Pantic, 2017; Puigvert et al., 2017; Díez-Palomar et al., 2018). This methodology has been implemented in previous and several competitive research projects addressed to identify communicative acts that promote NAM funded by the European Framework Program or the Spanish Plan for Scientific and Technical Research and Innovation, such as the project “Impact of the Communicative Acts and New Masculinities” (Soler, 2010-2012). It was selected because the CM is oriented toward social transformation by identifying both the components that represent barriers and those that contribute to the transformation of social inequalities. For this study, this approach allowed us to identify, on the one hand, the barriers related to language and communicative acts that can lead to the reproduction of DTM role by women and, on the other hand, the transformative elements related to the use of the language of desire by NAM that contributes to opposition to this sexist double standard and makes it a highly attractive alternative to DTM.

Also, the egalitarian dialogue is one of the principles which characterize CM, and it was convenient in this study for exploring aspects related to communicative acts on an issue as sensitive as intimate relationships. This egalitarian conception is shaped through the absence of interpretative hierarchies between participants in the research (Flecha, 2000; Oliver et al., 2011; Garcia Yeste et al., 2020). In CM, researchers participate in the dialogue by providing their own scientific knowledge and understanding, whereas researched subjects contribute equally with their daily life experiences and the knowledge acquired through living (Matulič-Domadzič et al., 2020; Rodríguez-Oramas et al., 2020). The use of the egalitarian dialogue in this study allowed the research team to share scientific evidence on the topic studied and to deepen the daily communicative stories from an egalitarian position. Throughout the dialogue, the interpretations of reality are jointly agreed upon in an intersubjective way.

### Participants and Data Collection Techniques

To promote an egalitarian dialogue between researchers and participants who recount their life experiences, a qualitative inquiry was conducted based on the communicative daily life story data collection technique (Ramis et al., 2014; Soler, 2015). This technique was designed to focus on dialogue about specific moments of a subject’s life. In this study, these moments account for the influence of communicative acts on heterosexual affective–sexual relationships and masculine identities. Through the communicative daily life stories technique, participants explained concrete situations, interactions, conversations, gestures, and other communicative acts that they have experienced in their life or with their friends or acquaintances. The egalitarian dialogue generated in the communicative daily life stories allowed us to gather cases and examples of interactions and communicative acts that lead to the reproduction of DTM role by women or of the communicative acts of NAM showing the use of a language of desire for rejecting to be the female *warrior’s rest*. At the beginning of the communicative daily life stories, researchers explained the purpose of the study, asking participants to feel free to delve more or less carefully into each experience. Researchers explained to them the theoretical foundations of the NAM approach as well as the myth of the *warrior’s rest*, and then, a dialogue was generated between researchers and participants around a script of general topics to be addressed. Participants contributed with their experience of this theory’s practical implications in terms of dialogues about relationships that illuminate these ideas. It is the foregrounding of the dialogue in these situations that the communicative daily life stories seek to elicit. The communicative daily life stories lasted between an hour and an hour and a half. They were conducted in Spanish, recorded, and later transcribed. Participants also signed informed consent. To ensure confidentiality and anonymity, all participants were given pseudonyms.

To reach and select participants, we employed snowball sampling. To answer to the aim of the study, we selected three men and two women because they present accounts related to the reproduction of the double standard and the myth of *warrior’s rest* or they provide essential information on these issues based on the experiences of their friends or acquaintances. These five persons narrate independently different experiences; that is, there were no connections between the situations described (Table 1). Researchers asked participants directly about their own or their friends’ experiences. Communicative daily life stories with women participants were focused primarily on how communicative acts among women can lead to the reproduction of the female *warrior’s rest* and double standards, whereas with men participants, the central theme addressed was NAM’s rejection toward the female *warrior’s rest* and the use of the language of desire in this process. However, both women and men participants shared experiences and communicative acts on both main themes.

**TABLE 1:** Participant profile.

Pseudonym	Gender	Age	Place of residence
Clara	Woman	30–35	Madrid
Maria	Woman	45–50	Burgos
Natalia	Woman	30–35	Madrid
Albert	Man	35–40	Catalonia
Eduard	Man	40–45	Catalonia

### Data Analysis

From this data set, we have included only responses that clearly demonstrate communicative acts and practices which reproduced the myth of *warrior’s rest* or challenged it. Through the communicative daily life stories and participants, we achieved to gather data on the following categories that respond to the aims of the study:

a) women who reproduced the myth of *warrior’s rest* and at the same time the traditional model of affective–sexual relationship based on the double standard;
b) women whose friends have also reproduced the myth of *warrior’s rest* and the double standard in their affective–sexual relationships;
c) men who have been involved in an affective–sexual relationship based on the myth of *warrior’s rest* and the traditional model of the double standard;
d) men whose circle of friends has been involved in an affective–sexual relationship based on the myth of *warrior’s rest* as well as the traditional model of the double standard; and
e) men who claimed they did not want to be *the second fiddle* to any woman.

We specifically analyze the role of communicative acts in social interaction to highlight the different types of language use and their effect on sexual–affective relationships. When analyzing the communicative acts, attention was paid to the situations that counted which elements of the communicative acts influenced that interaction, verbal communication, non-verbal communication, tone, gestures, if there was a power relationship, and the context in which the interaction took place, among others. Thereafter, the evidence and implications for the prevention of gender violence are explored.

## Results

The results are presented in two sections corresponding to each of the research questions. The first section focuses on exclusionary interactions that promote the double standard and reproduce the myth of *warrior’s rest*. The second section sheds light on the transformative use of language that goes beyond these two exclusionary elements: interactions and communicative acts which point to the existence of an alternative model to counteract this tendency, which has been defined in theory as the NAM (Flecha et al., 2013; Rodríguez-Navarro et al., 2014).

### Language and Interaction Among Women Promoting the Double Standard Toward Men

The information provided by the communicative daily life stories responds to the theoretical assumptions that some women imitate the male model of looking for a partner for the *warrior’s rest*. In that sense, when talking about the different models of attraction and relationship patterns, some of the interviewees report that women show this kind of behavior of attraction to men who treated them badly, causing them to have several unsatisfactory but sexually exciting relationships with this kind of men but then turn to non-dominant men to have a stable relationship and start a family life but lacking passion and sexual excitement. In order to respond to the first research question about *how language and social interaction among women can lead to the reproduction of esteem that DTM receive by women*, the analysis of the communicative daily life stories focuses on those communicative acts used among the participants or their circle of friends.

In this regard, during Maria’s daily life story talking about the diverse relationship patterns and the imitation of the *warrior’s rest* by one of her closest friends, she started to explain her relationship pattern, identifying similarities between her behavior and the aforementioned theoretical assumptions. According to what Maria’s friend explained to her, she repeats relationship patterns following the double standard, feeling attracted to men that according to Maria “are no good” and, on the other hand, choosing “nice guys” to settle down with but who bore her. In her narrative, Maria specifically mentions how her friend’s circle of friends influences this behavior, although Maria disapproves.

Maria: She has entered into a circle of friends, girls that are living this process and now among them they got angry at each other, and this world is so crazy that they even fight for the bad guys, you know?
Interviewer: And do you think that among them, they promote attraction to the bad guys?
Maria: So much! They are all the same! Because with these guys, when you tell them “these guys are no good for you,” “don’t believe everything they say” they don’t want to hear that so they establish a distance to those people who tell them [such things] and gather with their peers who encourage them.

In this contribution, Maria stresses the impact that her comments, in the form of advice, have on her friend’s desire toward “bad guys.” Instead of achieving Maria’s objective to change her friend’s mind about these kinds of men, more distance is created between Maria and her friend because of her questioning. This can be explained because her questioning is performed with a type of language which is only connected with ethical arguments, and this generates rejection as Maria’s friend prefers to talk about tastes and relationships with friends that currently promote her double standard-based practices. Thus, Maria’s explanation demonstrates that sometimes the use of language of ethics by saying “these guys are no good for you” is ineffective in preventing a woman from having relationships with them and can cause damaging effects.

In the same vein, although through a very different experience, Natalia’s story recalls interactions with her friends discussing the inversion of roles that one of her friends performs with “bad boys.” Thus, Natalia’s friend is having a stable relationship with a “nice guy” with whom she did not have sexual intercourse for a long time and is, at the same time, feeling attraction and sexual excitement for a man from their group who they identify as “the worst.” Natalia explains two kinds of interactions concerning her close friend. The first one refers to a specific interaction of that friend toward her partner in front of other friends and the effects of this attitude on him:

Natalia: The last days I spent at their house I felt kind of uncomfortable because she is really violent toward him, not physically but verbally. She ridicules him because they don’t have sex and does so in front of everybody. And that makes me feel very uncomfortable.
Interviewer: And what does she say?
Natalia: For example, she said, “well I’m happy you’re having sex, because we haven’t had sex for…,” turning to her boyfriend, “for how long didn’t we fuck, huh?” and it’s like “what the hell are you doing?!”
Interviewer: and how does he react to that?
Natalia: Oh he gets red and doesn’t say a word, or he gets into the tease and gets somehow aggressive and the rest just laugh with her!

The second interaction shows the conversations she had with that friend and other girls about the attraction and desire she feels toward the “bad guy,” even explicitly arguing because he is terrible.

Interviewer: And at the same time she tells you that she gets excited by the other guy?
Natalia: Yeah sure!
Interviewer: And how does she express that?
Natalia: Like, great “the other day I’ve been dancing with him, oh, and I got so excited! And I felt so guilty because it’s such a long time that I didn’t feel that way! And especially with him, who is the worst! I shouldn’t feel that way about him!” Well, pretty much like that. But in the group of friends there is another couple that is very nice, they are a bit older, like me and we tell her “this has nothing to do with love, the fact that this guy gets you excited” and we talk to her like that. But then she has other friends who say “Sure, that he gets you excited is love.” For example, Maria, who is with a musician and they have a relationship which is based on abuse but depends on the sexual excitation that she feels for being with a guy like that. So she understands and justifies that. (…) The first thing she said was “I knew that you would like this guy, as far as I know you, I knew it, so if you want to just let it flow and see where it ends, your partner won’t find out, he isn’t even in the same city.”

This part of the narrative provided by Natalia clearly evidences both the identification of the theory in real life with the double standard that the girl pursues in her relationships with men and how the interactions with her friends encourage her in reproducing this pattern of the *warrior’s rest*. It becomes clear that Natalia’s girlfriend’s humiliating behavior toward her partner is tolerated and appreciated by their friends. When Natalia’s friend asks her boyfriend in front of their friends for how long they had not had sexual intercourse, she is not merely posing a question. She is reproaching him for not having sex and ridiculing him as the person responsible for that situation. Whereas Natalia shows her rejection toward this behavior in the interview, according to her narrative, the friends seem to approve of this kind of behavior by laughing at her partner. This communicative act of laughing shows that the social practice of humiliating the partner is accepted and even emphasized since the person who humiliates finds approval for this in her circle of friends. According to the narrative, neither the partner who is humiliated nor the friends make any gesture of stopping this violent behavior expressed in the woman’s words. Despite the general acceptance of this situation, Natalia reports her discomfort with this kind of interactions and classifies them as violence. However, she does not report any communicative act expressed by any of the friends that would show this discomfort and counteract the behavior in question.

Moreover, Natalia also recalls how she and the group reacted to her friend’s crush on one of the “worst” guys from their circle of friends. While Natalia explains that she and another friend did not encourage her in progressing this affair, the narrative does not provide any evidence of communicative acts that would prevent her from engaging in this endeavor. Contrarily, these acts illustrate how some friends directly encourage her to reproduce the double standard and the pattern of the female *warrior’s rest*. Thus, by saying that she should continue with her affair while maintaining her partner’s relationship, she is being incited to cheat on her boyfriend.

Lastly, the narrative also clearly shows that the use of language refers to either model of masculinity that Natalia’s friend gets involved with. Whereas she ridicules her partner for whom she does not feel any sexual excitement in front of others, she expresses a desire for another man they consider as *the worst* for his dominant attitude. When explaining how her friend talks about this man, Natalia employed a tone of voice where the interviewer clearly identifies excitement and desire. At the same time, the sentence “the other day I’ve been dancing with him, oh, and I got so excited! And I felt so guilty because it’s such a long time that I didn’t feel that way! And especially with him, who is the worst! I shouldn’t feel that way about him!” shows the contradiction she experiences between what would be ethical and what she desires. It is precisely this contradiction which is promoted in the interactions with her friends, who encourage her to pursue the double standard, that is, leading her to continue to practice the pattern of the *warrior’s rest* in having a “good guy” as her stable partner and sexual excitement with “the worst guy” as an affair. Nonetheless, this role inversion and double standard reproduction are not always successful, particularly when men, who act following the premises of the NAM model, respond with a more principled reply. Several examples of this are presented in the following section.

### Guys Say It’s Enough! Moving Forward to Build the NAM

Eduard’s story illustrates some situations he experienced with an ex-girlfriend where he stopped her intention to transform him as the *warrior’s rest*, as he explained to his ex-girlfriend, who wanted a stable relationship to become a young mother and for that reason she desired a “good guy.” Her previous relationships mainly were with “bad boys,” but she was very anxious to get involved with a man that she could rapidly start a stable relationship.

Eduard: After 1 month of dating she asked me whether I loved her… and well she was in a hurry to run with that. But of course my reaction was “What are you doing?” right? I mean, “Your pushing an accelerator that doesn’t exist, there are no accelerators for that. Or it goes or it does not. But don’t think that everything goes as you want because you have already finished the puzzle and I am the missing piece in your puzzle.”

In Eduard’s relationship, there were different situations like this. In this regard, his ex-girlfriend continuously tried to confound him with scorning statements as an attempt to control his decisions. In the following quote, Eduard explains another of these situations.

Eduard: I am part of a men’s group and we meet monthly. So, we [he and his ex-girlfriend] met for the weekend, she came to sleep over on Friday, and we do the meetings on Saturdays and this time I had completely forgotten about it because we changed the date, we brought the date forward, I didn’t write it down, my fault, but she knew the importance it had to me. So I told her, that we couldn’t leave that weekend, that I was very sorry, I had mixed up the dates, but that we had the meeting and I couldn’t stop to go. She directly started to make a fuss, but a …. a huge fuss! (laughs) and I told her, also in other words, that I didn’t want that, that she should take her things and leave, that I didn’t want these kind of situations and less in my house, that she knew perfectly what makes me happy and to be honest that weekend, we didn’t have anything super important to do so that I couldn’t go to the meeting. So it was perfectly compatible.
Interviewer: And how did the situation end?
Eduard: Well she had an anxiety attack, fake, fake, she started to hyperventilate and you can see that she does so because … So I waited until it was over and I told her again to get it together and leave, that I didn’t want her to be there. But in the end it was late at night and I didn’t want her to be out on the streets on her own, so I took her home.

In his narrative, Eduard exemplifies his ex-girlfriend’s expectations toward the relationship and him, and when she saw that he would not exactly respond to her expectations, she started to get angry and act aggressively. In fact, the interactions described above evidence an adverse reaction in a given situation that his ex-girlfriend disliked. Eduard associates this reaction to his involvement in a men’s group where non-egalitarian myths such as the *warrior’s rest* are tackled. When the communicative acts of Eduard’s responses are analyzed, it is necessary to highlight his self-confidence in not permitting emotional blackmail and his decision to separate. Thus, it becomes clear that he rejects her behavior that, according to his interpretation, corresponds to her interest to transform him as her *warrior’s rest*.

The narrative further evidences that he keeps calm about the situation. He does not express any kind of disrespect toward her but, on the contrary, continues taking care of her and wanting the best for her. His communicative acts express that he rejects a certain behavior, and the verbal language used to express his rejection combines desire and ethics. In other words, he expresses his wish and desire for interactions free from violence by saying “I don’t want that” and by strengthening the force of this sentence with his gestures and actions of actually making her leave even when she tries to make him feel guilty about her physical condition through the anxiety attack.

A similar example is given by Clara, explaining one of her friends’ experience with his ex-girlfriend. When discussing the myth of the *warrior’s rest*, she recalls this relationship because, according to her, Laura, the ex-girlfriend of her friend Pau, shows behavioral traits that fit this description. Laura had been married, and when her dream of having children did not come true, she started to look for someone new and to recover the time she had “lost” being with the first and only man in her life. So she starts dating Pau, but when she sees that he is a good guy, Laura starts to have an affair with Juan, who is in a relationship himself and known to be disrespectful toward women and for having affairs constantly. When Pau finds out about this, he breaks up with Laura because he does not want this kind of relationship using similar communicative acts as those reported by Eduard.

Clara: He said “girl I think it’s great that you make out with anyone you like, maybe it’s not our time, enjoy it, and maybe in some years we find each other again and we’ll see what happens” because he wouldn’t cut the wings of nobody. So he told her “I don’t want this for me, I don’t like this,” the fact that one person, because he found out through the chat, because she had left it open and he saw the conversation she had with Juan, the dog trainer, so he found out about it. (…)

As a result, the conversation with Clara and especially the description of Pau, contrasted with the description of Juan, are interesting to analyze in terms of the type of language that she employs to describe Pau, which is full of desire evoking an attractive image of Pau.

Clara: She [the ex-girlfriend] had broken up her previous relationship because she was married and she saw Pau as something new, someone good, but he has this feature of the unknown, he likes to go to the mountains, to take out the dogs, he knows people, like something different to what she had before, but when she sees that in spite of all this he is a good guy, because he is a good guy and he is a super fair person, like … he is a fucking great guy! you know? A super just guy, egalitarian and so on. And she went to look for Juan who is the opposite, a chauvinist, a liar, taking advantage of everyone, and so on.

As Clara’s words show, describing Pau as a “fucking great guy,” as well as with the tone of voice she employed in the interview when she talks about him, a language use that mixes desire and respect is evidenced. In the first quote, Pau knows his worth, and he does not tolerate being with a woman who only wants him for a stable relationship without any passion, as it can be observed in his words: “maybe it’s not our time, enjoy it” and “I don’t want this for me, I don’t like this.” That is what Clara emphasizes in her narrative and what she values about Pau.

The communicative acts analyzed in the present section evidence their effect on women when men, following some premises of the NAM model, use language to demonstrate they are not playing second fiddle to anybody. Particularly, findings suggest how performing a language of desire challenges the chauvinist double standards that some women imitate is becoming crucial to increasing these men’s sex appeal. In other words, this language generates attraction and excitement because it is especially based on self-confidence as well as coherence between the words and the feelings that men exhibit by rejecting being a female *warrior’s rest*.

## Discussion

The present research adds new knowledge through the analysis of communicative acts and masculinities, evidencing the importance of language uses in reproducing the double standards in gender relations. Although some previous analyses confirmed how the traditional double standard is reproduced from a language of desire which promotes dominant masculinities (Flecha et al., 2013; López de Aguileta et al., 2020), there are no deeper analysis on how this language maintains this double standard throughout practices as the myth of *warrior’s rest* implies. Scientific literature has also confirmed that some women reproduce this double standard by imitating this myth (Lyons et al., 2011) and by how they choose “nice guys” for stable relationships and “macho men” for having “fun” and sexual encounters (Herold and Milhausen, 1999; Urbaniak and Kilmann, 2003; McDaniel, 2005; Ahmetoglu and Swami, 2012). However, none of these analyses study in detail the role of interactions, conversations, and non-verbal language in the perpetuation of this conventional male practice. Thus, our analysis contributes to a focus on this gap on the influence of people’s communication on the shaping of this myth.

The second contribution of the article refers to the importance of communicative acts and their influence on partner selection among men (Renninger et al., 2004; Brak-Lamy, 2015; Fisher et al., 2020). The present research provides a novel insight into the potential of the use of language to break the tendency of reproducing the double standard in heterosexual relationships and the model of the female warrior. The evidence of language use contributes to emphasizing the distinct features of alternative masculinities to the hegemonic model and represents a theoretical model of masculinity that can contribute to gender violence prevention. In this regard, the present research shows that some men use communicative acts to overcome a pattern of female behaviors based on the dominant OTM or being dominated by DTM. With a language of desire, these men do not allow oppression and domination by women who behave as female *warriors* but show their rejection of this kind of behavior. Both of the above contributions should be examined considering some limitations regarding access to suitable participants willing to provide information on the sensitive themes addressed, which may have limited the data obtained. Another limitation is related to relying on participants’ self-reported data that may involve potential limitations regarding selective memory when recalling past events, together with attributing events and outcomes to lived situations and interactions experienced with other people. Nevertheless, the findings presented have several future implications for research and educational interventions to address gender violence. Concerning research, there is a dearth of investigations that focus their attention on the importance of language in the reproduction of traditional practices, like the myth of *warrior’s rest*, where female attraction to “bad boys” is encouraged or supported. Therefore, this is a research field that could be widened in order to understand how and why these practices are maintained and which kind of languages use can contribute to preventing them. Referring to interventions, the results presented in the article can help to construct educational actions and to design social policies where the language of desire and NAM become a central focus of educational practice for gender equality and liberation from patriarchal assumptions and hegemonic practices.

## Data Availability Statement

The raw data supporting the conclusions of this article will be made available by the authors, without undue reservation.

## Ethics Statement

All participants were informed that their participation was anonymous and voluntary and that data would be treated confidentially and used for research purposes only. Ethical requirements were addressed following the Ethical Review Procedure established by the European Commission (2013) for EU research, the Data Protection Directive 95/46/EC, and the Charter of Fundamental Rights of the European Union (2000/C 364/01). This research was reviewed and approved by the Ethics Committee of the Community of Research on Excellence for all.

## Author Contributions

LR-E performed the conceptualization. LR-E and GM conducted the research and investigation process. LR-E, GM, and AT conducted the data analysis. LR-E, GM, and AT prepared the original draft. LR-E and JC revised the draft critically for important intellectual content. All authors have read and agreed to the published version of the manuscript.

## Conflict of Interest

The authors declare that the research was conducted in the absence of any commercial or financial relationships that could be construed as a potential conflict of interest.

## References

[B1] Ahmetoglu G. Swami V. (2012). Do Women Prefer “Nice Guys? The Effect of Male Dominance Behavior on Women’s Ratings of Sexual Attractiveness. *Soc. Behav. Pers.* 40 667–672. 10.2224/sbp.2012.40.4.667

[B2] Álvarez-Muelas A. Gómez-Berrocal C. Sierra J. C. (2021). Typologies of sexual double standard adherence in Spanish population. *Eur. J. Psychol. Appl. Leg. Context* 13 1–7. 10.5093/ejpalc2021a1

[B3] Amaro H. D. Alvarez M. J. Ferreira J. A. (2020). Portuguese College Students’. Perceptions About the Social Sexual Double Standard: developing a Comprehensive Model for the Social SDS. *Sex. Cult.* 25 733–755. 10.1007/s12119-020-09791-9

[B4] Armstrong J. Thorpe S. Williams D. (2020). Sexual Attitudes. Religious Commitment, and Sexual Risk Behaviours among College-Aged Women: a Feminist Theory Approach. *J. Gend. Stud.* 2020 1–12. 10.1080/09589236.2020.1838888

[B5] Arnold L. (2019). Language socialization across borders: producing scalar subjectivities through material-affective semiosis. *Pragmatics* 29 332–356. 10.1075/prag.18013.arn 33486653

[B6] Austin J. L. (1962). *How To Do Things With Words.* Oxford: Oxford university press.

[B7] Barros del Río M. A. (2005). How to Disguise Fairy Tales in 21st Century Ireland. A Feminist Analysis of Marian Keyes’ and Cathy Kelly’s Blockbusters *Estudios Irlandeses* 2005 12–21. 10.24162/ei2005-640

[B8] Baxter J. (2006). *Speaking Out in Public Contexts. Basingstoke.* London: Palgrave.

[B9] Beauvoir S. (1949). *The Second Sex.* United States: Vintage Books.

[B10] Boulton J. (2020). Of friends and kollegen: understanding male friendships in Swakopmund, Namibia. *Anthropol. South. Africa* 43 169–180. 10.1080/23323256.2020.1754130

[B11] Brak-Lamy G. (2015). Heterosexual seduction in the urban night context: behaviors and meanings. *J. Sex Res.* 52 690–699. 10.1080/00224499.2013.856835 24483605

[B12] Bridges T. Pascoe C. J. (2014). Hybrid masculinities: new directions in the sociology of men and masculinities. *Sociol. Compass* 8 246–258. 10.1111/soc4.12134

[B13] Burdelski M. (2013). “I’m sorry, flower”: socializing apology, relationships, and empathy in Japan. *Pragmat. Soc.* 4 54–81. 10.1075/ps.4.1.03bur 33486653

[B14] Connell R. (1995). *Masculinities.* Berkeley: University of California Press.

[B15] Connell R. (1987). *Gender and power: Society, the person, and sexual politics.* Stanford, CA: Stanford University Press.

[B16] da Luz Scherf E. de Lourdes Alves, Lima Zanatta M. Mello E. T. (2020). Gender-Awareness amongst Brazilian Children Enrolled in Primary Education: implications for Women’s Rights & Equality. *Multidiscip. J. Gend. Stud.* 9 25–50. 10.17583/generos.2020.4865

[B17] Desrochers S. (1995). What types of men are most attractive and most repulsive to women? *Sex Roles* 32 375–391. 10.1007/BF01544603

[B18] Díez-Palomar J. Capllonch M. Aiello E. (2014). Analyzing Male Attractiveness Models From a Communicative Approach: socialization, Attraction, and Gender-Based Violence. *Qual. Inq.* 20 844–849. 10.1177/1077800414537205

[B19] Díez-Palomar J. Sanmamed A. F. F. García-Carrión R. Molina-Roldán S. (2018). Pathways to Equitable and Sus-tainable Education through the Inclusion of Roma Students in Learning Mathematics. *Sustainability* 10:2191. 10.3390/su10072191

[B20] Dunn H. K. Gjelsvik A. Pearlman D. N. Clark M. A. (2014). Association between Sexual Behaviors. Bullying Victimization and Suicidal Ideation in a National Sample of High School Students: implications of a Sexual Double Standard *Womens Health Issues* 24 567–574. 10.1016/j.whi.2014.06.008 25213749

[B21] Duque E. Rodríguez-Conde J. Puigvert L. Peña-Axt J. C. (2020). Bartenders and Customers’. Interactions. Influence on Sexual Assaults in Nightlife *Sustainability* 12 6111. 10.3390/su12156111

[B22] Fabius S. (2005). Homosocial desire in men’s talk: balancing and re-creating cultural discourses of masculinity. *Lang. Soc.* 34 695–726.

[B23] Fairclough N. (2003). Critical Applied Linguistics: a critical introduction. *Discourse Soc.* 14 805–808. 10.1177/09579265030146008

[B24] Fairclough N. (2006). *Language and Globalization.* London: Routledge.

[B25] Fisher M. L. Coughlin S. Wade T. J. (2020). Can I have your number? Men’s perceived effectiveness of pick-up lines used by women *Pers. Individ. Dif.* 153:109664. 10.1016/j.paid.2019.109664

[B26] Flecha A. Puigvert L. (2010). “Contributions to Social Theory from Dialogic Feminism: Giving a Voice to All Women,” in *Examining social theory: Crossing borders/reflecting back*, ed. Chapman P. (New York: Peter Lang), 161–174.

[B27] Flecha R. (2000). *Sharing Words. Theory and Practice of Dialogic Learning.* Lanham: Rowman & Littlefield.

[B28] Flecha R. (2014). Using Mixed Methods From a Communicative Orientation Researching With Grassroots Roma. *J. Mix. Methods Res.* 8 245–254. 10.1177/1558689814527945

[B29] Flecha R. Puigvert L. Ríos O. (2013). The New Alternative Masculinities and the Overcoming of Gender Violence. *Int. Multisciplin. J. Soc. Sci.* 2 88–113.

[B30] Flecha R. Tomás G. Vidu A. (2020). Contributions from psychology to effective use and achievement of sexual consents. *Front. Psychol.* 11:92. 10.3389/fpsyg.2020.00092 32140122 PMC7042399

[B31] Garcia Yeste C. El Miri O. Álvarez P. Morlà T. (2020). Muslim women wearing the niqab in Spain: dialogues around discrimination, identity and freedom. *Int. J. Intercult. Relat.* 75 95–100. 10.1016/j.ijintrel.2020.02.003

[B32] Georgakopoulou A. (2005). Styling men and masculinities: interactional and identity aspects at work. *Lang. Soc.* 34 163–184.

[B33] Giordano P. C. Longmore M. A. Manning W. D. (2006). Gender and the Meanings of Adolescent Romantic Relationships: a focus boys. *Am. Sociol. Rev.* 71 260–287. 10.1177/000312240607100205

[B34] Gómez A. (2014). How Friendship Generates Key Research Questions That Help to Overcome Gender-Based Violence: a Personal Narrative. *Qual. Inq.* 20 934–940. 10.1177/1077800414537220

[B35] Gómez A. (2017). “Communicative methodology and social impact,” in *Qualitative Inquiry in Neoliberal Times*, eds Denzin N. K. Giardina M. D. (New York: Routledge), 166–178. 10.4324/9781315397788-12

[B36] Gómez A. Padrós M. Ríos O. Mara L. C. Pukepuke T. (2019). Reaching social impact through communicative methodology. Researching with rather than on vulnerable populations: the Roma case. *Front. Educ.* 4 1–9. 10.3389/feduc.2019.00009

[B37] Gómez A. Puigvert L. Flecha R. (2011). Critical Communicative Methodology: informing real social transformation through research. *Qual. Inq.* 17 235–245. 10.1177/1077800410397802

[B38] Gómez A. Siles G. Tejedor M. (2012). Contribuir a la transformación social a través de la Metodología de Investigación Comunicativa. *Qual. Res. Educ.* 1 36–57.

[B39] Gómez J. (2015). *Radical Love: A revolution for the 21st century.* New York: Peter Lang Publishing.

[B40] Gouda-Vossos A. Nakagawa S. Dixson B. J. Brooks R. C. (2018). Mate choice copying in humans: a systematic review and meta-analysis. *Adapt. Human Behav. Physiol.* 4 364–386. 10.1007/s40750-018-0099-y

[B41] Greer G. (1970). *The Female Eunuch.* New York: Harper Perennial Modern Classics.

[B42] Habermas J. (1987). *Teoría de la acción comunicativa. Vol. I-II.* Madrid: Taurus.

[B43] Hatz L. E. Park S. McCarty K. N. McCarthy D. M. Davis-Stober C. P. (2020). Young adults make rational sexual decisions. *Psychol. Sci.* 31 944–956. 10.1177/0956797620925036 32783528 PMC7580154

[B44] Hawley P. Hensley W. A. (2009). Social Dominance and Forceful Submission Fantasies: femenine Pathology or Power?. *J. Sex Res.* 46 568–585. 10.1080/00224490902878985 19353371

[B45] Herold E. Milhausen R. (1999). Dating preferences of university women: an analysis of the nice guy stereotype. *J. Sex Marital. Ther.* 25 333–343. 10.1080/00926239908404010 10546171

[B46] Elboj-Saso C. Iñiguez-Berrozpe T. Valero-Errazu D. (2020). Relations With the Educational Community and Transformative Beliefs Against Gender-Based Violence as Preventive Factors of Sexual Violence in Secondary Education. *J. Interpers. Violence* 088626052091364. 10.1177/0886260520913642 [Online ahead of print] 32253970

[B47] Jackson S. (2018). Young feminists, feminism and digital media. *Fem. Psychol.* 28 32–49. 10.1177/0959353517716952

[B48] Killoren S. E. Campione-Barr N. M. Jones S. K. Giron S. E. (2019). Adolescent Girls’. Disclosure About Dating and Sexuality. *J. Fam. Issues* 40 887–910. 10.1177/0192513X19829501

[B49] Kim H. S. (2020). Sexual debut and college entrance among South Korean adolescents. *J. Adolesc.* 85 126–134. 10.1016/j.adolescence.2020.11.004 33227598

[B50] Kreager D. A. Staff J. (2009). The Sexual Double Standard and Adolescent. *Soc. Psychol. Q.* 72 143–164. 10.1177/019027250907200205 25484478 PMC4256532

[B51] Little A. C. Caldwell C. A. Jones B. C. DeBruine L. M. (2015). Observer age and the social transmission of attractiveness in humans: younger women are more influenced by the choices of popular others than older women. *Br. J. Psychol.* 106 397–413. 10.1111/bjop.12098 25314951

[B52] López de Aguileta G. Torras-Gómez E. García-Carrión R. Flecha R. (2020). The emergence of the language of desire toward nonviolent relationships during the dialogic literary gatherings. *Lang. Educ.* 34 1–16. 10.1080/09500782.2020.1801715

[B53] Lyons H. Giordano P. C. Manning W. D. Longmore M. A. (2011). Identity, Peer Relationships, and Adolescent Girls’. Sexual Behavior: An Exploration of the Contemporary Double Standard. *J. Sex Res.* 48 437–449. 10.1080/00224499.2010.506679 20818574 PMC3101306

[B54] Manago A. M. Ward L. M. Aldana A. (2015). The sexual experience of Latino young adults in college and their perceptions of values about sex communicated by their parents and friends. *Emerg. Adulthood* 3 14–23. 10.1177/2167696814536165

[B55] Mañas-Viejo C. Martínez Sanz A. (2020). Between Coercion and Consent: a Study on Male Sexual Violence in Heterosexual Partner Relationships. *Masculinities Soc. Chan.* 6 235–260. 10.17583/MCS.2020.5663

[B56] Matulič-Domadzič V. Munté-Pascual A. De Vicente-Zueras I. León-Jiménez S. (2020). “Life Starts for Me Again”. The Social Impact of Psychology on Programs for Homeless People: solidarity Networks for the Effectiveness of Interventions. *Front. Psychol.* 10:3069. 10.3389/fpsyg.2019.03069 32116874 PMC7010906

[B57] McClintock E. A. (2011). Handsome wants as handsome does: physical attractiveness and gender differences in revealed sexual preferences. *Biodemography. Soc. Biol.* 57 221–257. 10.1080/19485565.2011.615172 22329089

[B58] McDaniel A. (2005). Young Women’s Dating Behavior: why/Why not Date a Nice Guy? *Sex Roles* 53 348–359. 10.1007/s11199-005-6758-z

[B59] Mensah E. O. (2021). To be a Man is Not a Day’s Job: the Discursive Construction of Hegemonic Masculinity by Rural Youth in Nigeria. *Gend. Issues* 2021 1–23. 10.1007/s12147-020-09271-2

[B60] Migliaccio T. (2014). Typologies of Men’s Friendships: constructing Masculinity through Them. *Masculinities Soc. Chan.* 3 119–147. 10.17583/msc.2014.1073

[B61] Mullaney J. L. (2007). Telling It Like a Man. *Men Masculinities* 10 222–247. 10.1177/1097184x06287758

[B62] Oliver E. de Botton L. Soler M. Merrill B. (2011). Cultural intelligence to overcome educational exclusion. *Qual. Inq.* 17 267–276. 10.1177/1077800410397805

[B63] Pantic N. (2017). Reconciling rigour and impact by collaborative research design: study of teacher agency. *Int. J. Res. Method Educ.* 40 329–344. 10.1080/1743727X.2015.1113250

[B64] Pecheny M. Zaidan L. Lucaccini M. (2019). Sexual activism and “actually existing eroticism”: the politics of victimization and “lynching” in Argentina. *Int. Sociol.* 34 455–470. 10.1177/0268580919854297

[B65] Perilloux C. Cloud J. M. Buss D. M. (2013). Women’s physical attractiveness and short-term mating strategies. *Pers. Individ. Dif.* 54 490–495. 10.1016/j.paid.2012.10.028

[B66] Portell D. Pulido C. (2012). Communicative Acts Which Promote New Masculinities. Overcoming Hegemonic Masculinity in the Workplace and the School. *Masculinities Soc. Chan.* 1 61–80. 10.17583/msc.2012.159

[B67] Price M. E. Pound N. Dunn J. Hopkins S. Kang J. S. (2013). Body Shape Preferences: associations with Rater Body Shape and Sociosexuality. *PLoS One* 8:e52532. 10.1371/journal.pone.0052532 23300976 PMC3534680

[B68] Puigvert L. Gelsthorpe L. Soler-Gallart M. Flecha R. (2019). Girls’ perceptions of boys with violent attitudes and behaviours, and of sexual attraction. *Palgrave Commun.* 5 1–12. 10.1057/s41599-019-0262-5

[B69] Puigvert L. Valls R. Garcia Yeste C. Aguilar C. Merrill B. (2017). Resistance to and Transformations of Gender-Based Violence in Spanish Universities: a Communicative Evaluation of Social Impact. *J. Mix. Methods Res.* 13:155868981773117. 10.1177/1558689817731170

[B70] Racionero-Plaza S. Duque E. Padrós M. Molina-Roldán S. (2021). “Your Friends Do Matter”: peer Group Talk in Adolescence and Gender Violence Victimization. *Children* 8:65. 10.3390/children8020065 33498532 PMC7909546

[B71] Ramis M. Martin N. Íñiguez T. (2014). How the Dialogue in Communicative Daily Life Stories Transforms Women’s Analyses of Why They Suffered Gender Violence. *Qua. Inq.* 20 876–882. 10.1177/1077800414537210

[B72] Renninger L. A. Wade T. J. Grammer K. (2004). Getting that female glance: patterns and consequences of male nonverbal behavior in courtship contexts. *Evol. Hum. Behav.* 25 416–431. 10.1016/j.evolhumbehav.2004.08.006

[B73] Rios-González O. Peña Axt J. C. Duque Sanchez E. De Botton Fernández L. (2018). The language of ethics and double standards in the affective and sexual socialization of youth Communicative acts in the family environment as protective or risk factors of intimate partner violence. *Front Sociol.* 3:19. 10.3389/fsoc.2018.00019

[B74] Rodríguez-Navarro H. Ríos-González O. Racionero S. Macías F. (2014). New methodological insights into communicative acts that promote new alternative masculinities. *Qual. Inq.* 20 870–875. 10.1177/1077800414537209

[B75] Rodríguez-Oramas A. Zubiri H. Arostegui I. Serradell O. Sanvicén-Torné P. (2020). Dialogue With Educators to Assess the Impact of Dialogic Teacher Training for a Zero-Violence Climate in a Nursery School. *Qual. Inq.* 26 1019–1025. 10.1177/1077800420938883

[B76] Searle J. (1998). *Mind, language & society: Philosophy in the real world.* New York: Basic Books.

[B77] Searle J. Soler M. (2004). *Lenguaje y ciencias sociales. Diálogo entre John Searle y CREA [Language and Social Sciences. Dialogue between John Searle and CREA].* Barcelona: El Roure.

[B78] Soler M. (2015). Biographies of “Invisible”. People Who Transform Their Lives and Enhance Social Transformations Through Dialogic Gatherings. *Qual. Inq.* 21 839–842. 10.1177/1077800415614032

[B79] Soler M. (2010-2012). *Impacto de los actos comunicativos y nuevas masculinidades [Impact of communicative acts and new masculinities].* Madrid: Ministry of Science and Innovation. 10.1177/1077800415614032

[B80] Stobbe L. (2005). Doing machismo: legitimating speech acts as a selection discourse. *Gend. work Organ.* 12 105–123. 10.1111/j.1468-0432.2005.00265.x

[B81] Tifft E. (2015). “The Case for the “Nice Guy”: Why the Collegiate Reign of “Bad Boys” is a Feminist Disaster—and How Nice Guys Bring about Women’s Empowerment” in*The Elizabeth Cady Stanton Student Research Conference Proceedings (Vol. 1).* New Jersey: Rutgers University Press

[B82] Torras-Gómez E. Puigvert L. Aiello E. Khalfaoui A. (2020). Our Right to the Pleasure of Falling in Love. *Front. Psychol.* 10:3068. 10.3389/fpsyg.2019.03068 32038418 PMC6987459

[B83] Urbaniak G. C. Kilmann P. R. (2003). Physical Attractiveness and the “Nice Guy Paradox”: do Nice Guys Really Finish Last? *Sex Roles* 49 413–426. 10.1023/A:1025894203368

[B84] Valls R. Puigvert L. Duque E. (2008). Gender Violence Amongst Teenagers: socialization and Prevention. *Violence Against Women* 14 759–785. 10.1177/1077801208320365 18559866

[B85] Wells T. Baguley T. Sergeant M. Dunn A. (2013). Perceptions of Human Attractiveness Comprising Face and Voice Cues. *Arch. Sex. Behav.* 42 805–811. 10.1007/s10508-012-0054-0 23358855

[B86] White K. P. Jonason P. K. Al-Shawaf L. (2020). Mating Decisions in the Absence of Physical Attraction. *Adapt. Hum. Behav. Physiol.* 7 43–53. 10.1007/s40750-020-00152-2

[B87] Wright B. L. Sinclair H. (2012). Pulling the strings: effects of friend and parent opinions on dating choices. *Pers. Relationsh.* 19 743–758. 10.1111/j.1475-6811.2011.01390.x

[B88] Zaikman Y. Marks M. J. (2014). Ambivalent Sexism and the Sexual Double Standard. *Sex Roles* 71 333–344. 10.1007/s11199-014-0417-1

