# Corrigendum: Making Choices in Discourse: New Alternative Masculinities Opposing the “Warrior's Rest”

**DOI:** 10.3389/fpsyg.2021.736545

**Published:** 2021-07-28

**Authors:** Laura Ruiz-Eugenio, Ana Toledo del Cerro, Jim Crowther, Guiomar Merodio

**Affiliations:** ^1^Department of Theory and History of Education, University of Barcelona, Barcelona, Spain; ^2^Department of Sociology, University of Barcelona, Barcelona, Spain; ^3^Moray House School of Education and Sport, IECS, University of Edinburgh, Edinburgh, United Kingdom; ^4^Department of Education, Nebrija University, Madrid, Spain

**Keywords:** warrior's rest, double standards, communicative acts, new alternative masculinities, the language of desire

In the published article, there were two errors in affiliations 2 and 4. The affiliations 2 and 4 are interchanged. Instead of “Department of Education, Nebrija University, Madrid, Spain” for affiliation 2, it should be “Department of Sociology, University of Barcelona, Barcelona, Spain.” Also, Instead of “Department of Sociology, University of Barcelona, Barcelona, Spain” for affiliation number 4 it should be “Department of Education, Nebrija University, Madrid, Spain.”

In the published article, there is an error in the correspondence email. Instead of “guiomar.merodio@gmail.com” it should be, gmerodio@nebrija.es.

The authors apologize for this error and state that this does not change the scientific conclusions of the article in any way. The original article has been updated.

## Publisher's Note

All claims expressed in this article are solely those of the authors and do not necessarily represent those of their affiliated organizations, or those of the publisher, the editors and the reviewers. Any product that may be evaluated in this article, or claim that may be made by its manufacturer, is not guaranteed or endorsed by the publisher.

